# Analysis of Risk Factors for Perioperative Acute Kidney Injury and Management Strategies

**DOI:** 10.3389/fmed.2021.751793

**Published:** 2021-12-24

**Authors:** Xiang Yu, Zhe Feng

**Affiliations:** State Key Laboratory of Kidney Diseases, Department of Nephrology, National Clinical Research Center of Kidney Diseases, Chinese PLA Institute of Nephrology, Chinese PLA General Hospital, Beijing, China

**Keywords:** AKI, perioperative, risk factors, prevention, management

## Abstract

Acute kidney injury (AKI) is a serious clinical syndrome, and one of the common comorbidities in the perioperative period. AKI can lead to complications in surgical patients and is receiving increasing attention in clinical workup. In recent years, the analysis of perioperative risk factors has become more in-depth and detailed. In this review, the definition, diagnosis, and pathophysiological characteristics of perioperative AKI are reviewed, and the main risk factors for perioperative AKI are analyzed, including advanced age, gender, certain underlying diseases, impaired clinical status such as preoperative creatinine levels, and drugs that may impair renal function such as non-steroidal anti-inflammatory drugs (NASIDs), ACEI/ARB, and some antibiotics. Injectable contrast agents, some anesthetic drugs, specific surgical interventions, anemia, blood transfusions, hyperglycemia, and malnutrition are also highlighted. We also propose potential preventive and curative measures, including the inclusion of renal risk confirmation in the preoperative assessment, minimization of intraoperative renal toxin exposure, intraoperative management and hemodynamic optimization, remote ischemic preadaptation, glycemic control, and nutritional support. Among the management measures, we emphasize the need for careful perioperative clinical examination, timely detection and management of AKI complications, administration of dexmedetomidine for renal protection, and renal replacement therapy. We aim that this review can further increase clinicians' attention to perioperative AKI, early assessment and intervention to try to reduce the risk of AKI.

## Introduction

Acutekidney injury (AKI) is a common clinical syndrome characterized by a sudden and persistent decline in kidney function over hours to days. It has numerous etiologies and complex mechanisms. AKI has received increasing attention from clinicians as one of the major perioperative comorbidities. Studies have reported the incidence of in-hospital AKI to be 2–18% in perioperative patients and 22–57% in intensive care patients (1). Perioperative AKI can lead to increased mortality, incidence of chronic kidney disease (CKD) and risk of postdischarge hemodialysis (HD), as well as increased healthcare costs and resource utilization (2, 3). Therefore, the concept of perioperative AKI should be explored in depth, and the associated risk factors should be analyzed to enhance the prediction and assessment of AKI, intervene in advance, and try to reduce the severity of AKI when it occurs.

## Definition and Diagnosis of Perioperative AKI

Perioperative AKI is defined as an acute decline in renal function occurring from 5–7 d before to 7-12 d after surgery (4), but there is no standard definition of AKI, resulting in a lack of clarity in clinical workup. The consensus for AKI was set in the 2002 RIFLE criteria with reference to sCr values and urine volume. The revised RIFLE criteria were published by AKIN in 2005, the main changes being the recognition that small changes in sCr values may increase morbidity and mortality and allowing AKI to be defined without knowledge of baseline sCr. In 2012, KDIGO proposed clinical practice guidelines for AKI, giving an updated definition of blood creatinine ≥26.5 mmol/L within 48 h, blood creatinine ≥1.5 times the basal level within 7 d or urine output <0.5 mL/kg/h for 6 h (5) (Table 1). However, these criteria still have drawbacks. First, the perioperative period has been shown to be a unique environment where stress and hypovolemia cause frequent intraoperative and postoperative reductions in urine output. During surgery, only 5–15% of the crystalloid volume load is excreted from the urine, compared to 40–75% in non-anesthetized patients (6, 7). The same is true for sCr, which has a relative lag, and it changes after 50% of the kidney function is impaired. Serum creatinine may also be influenced by a variety of factors, including ethnicity, age, sex, chronic disease, nutrition, volume overload, body mass, and muscle trauma (8).

**Table 1:** AKI diagnostic criteria.

**Staging**	**Urine volume**	**Diagnostic criteria**		
		**RIFLE (2002)**	**AKIN (2005)**	**KIDGO (2012)**
1 issue	Urine output <0.5 ml/kg/h > 6 h	**Risk:** sCr elevated > 1.5 times or GFR decline > 25%	sCr elevated ≥ 26.4 μmol/L or elevated > 1.5–2.0 times the baseline value	sCr elevated ≥ 26.5 μmol/L (0.3 mg/dl) or elevated > 1.5–1.9 times the baseline value
2 issues	Urine output <0.5 ml/kg/h > 12 h	**Injury:** sCr elevated > 2 times or GFR decline > 50%	sCr elevation > 2.0–3.0 times the baseline value	Elevation > 2.0–2.9 times the baseline value
3 issues	Urine output <0.3 ml/kg/h > 24 h or no urine for >12 h	**Failure:** sCr elevated > 3 times or GFR decline > 75% sCr ≥ 354 μmol/L or an acute increase of ≥ 44 μmol/L **Loss:** persistent renal failure > 4 weeks **ESRD:** persistent renal failure > 3 months	sCr elevation > 3.0 times the baseline value or sCr value ≥ 354 μmol/L or an increase in acute sCr values ≥ 44 μmol/L	sCr elevated ≥353.6 μmol/L (4 mg/dl) or elevated > 3.0 times the baseline value or starting kidney replacement therapy or <18-year-old patients with a decrease in eGFR to <35 ml/min/1.73 m^2^

Recent research on biomarkers that contribute to the early diagnosis of AKI has made important advances. Target biomarkers require that they be released and detectable in blood or urine before an increase in sCr and/or a decrease in urine output, with the aim of detecting subclinical AKI before kidney tissue damage and functional decline (9, 10). Different markers may be associated with different parts of the kidney and different mechanisms of injury, so the detection of AKI biomarkers not only allows the early detection of subclinical AKI but also helps to determine the cause and implement specific treatment. The functional markers neutrophil gelatinase-associated lipoprotein (NGAL) and liver-fatty acid binding protein (L-FABP) have been approved for use in Europe and Japan, respectively, while the combination of the cell cycle markers tissue inhibitor of metalloproteinase 2 (Timp-2) and insulin-like growth factor binding protein-7 (IGFBP7) have also been approved by the US FDA (11). Other target molecules include kidney injury molecule-1 (KIM-1), interleukin 18 (IL-18), calprotectin (CALPRO), β2-microglobulin (β2-MG), total urinary protein (UTP), microRNAs, etc (12–17). These markers have been well-studied, but their clinical predictive power is still unsatisfactory, and studies are being conducted with artificial intelligence to construct AKI prediction models, which will be more rational and effective when applied along with biomarkers as important parameters.

## Pathophysiology

Inadequate perfusion is the main mechanism affecting renal function, and all causes of decreased mean arterial pressure (MAP) can lead to renal hypoperfusion. Initially, the kidney can maintain GFR by activating the sympathetic nervous system, including the release of vasopressin (ADH) and angiotensin II (ANG II), activating the renin-angiotensin-aldosterone system (RAAS), with the end result of water and sodium retention to maintain GFR. Next, if the renal perfusion deficit is not corrected in time, angiotensin II eventually causes vasoconstriction of afferent and efferent small arteries, which decreases GFR. These mechanisms work only if MAP is kept above the autoregulatory threshold; below 75–80 mmHg, the efficiency of autoregulation decreases sharply.

Along with a deeper understanding of the mechanisms of AKI occurrence, it was found that a purely ischemic model does not explain sepsis and major surgery-related AKI well. The hemodynamics of sepsis can be predominantly hyperdynamic, and overall renal blood flow may remain constant or increase, but the glomerular filtration rate decreases significantly. In addition, perioperative AKI is caused not by a single factor but by multiple insults. Reduced perfusion may not be the only cause of surgery-related AKI, as partial occlusion of the renal artery for a certain period of time can also be tolerated by the kidney, and it is now believed that systemic inflammatory response syndrome (SIRS) is a non-specific response of the body to surgical trauma through microcirculatory dysfunction, intrinsic repair mechanism abnormalities, inflammation- and immune-mediated injury, and endothelial cell dysfunction, resulting in a combined response that leads to AKI (18).

## Risk Factors

### Impaired Clinical Status

Along with advances in surgical techniques, anesthesia techniques, and related care monitoring technologies, surgery in high-risk elderly patients is being performed more and more often. Comorbidities, acute illnesses and age-related decline in physiological reserve have led to an increased risk of perioperative AKI in surgical patients. From the large amount of prospective data on surgical patients, the identifiable risk factors for AKI are age ≥56 years, male sex, active congestive heart failure, ascites, hypertension, preoperative creatinine >106 mol/L, diabetes mellitus (controlled by oral medications or insulin injections) (19–24), and patients with six or more risk factors have an increased incidence and hazard ratio of AKI (25). Additional risk factors include ventilator dependence, chronic obstructive pulmonary disease, smoking, coagulation disorders, cancer, obesity, and long-term steroid medication use (26, 27) (Table 2).

**Table 2:** Analysis of risk factors for perioperative AKI.

**Risk factors**	**Details of contents**		
Impaired clinical status	Age ≥ 56 years, male, active congestive heart failure, ascites, hypertension, preoperative creatinine > 10^6^ mol/L, diabetes mellitus (controlled by either oral medication or insulin injections), ventilator dependence, chronic obstructive pulmonary disease, smoking, coagulation disorders, cancer, obesity, and long-term steroid use		
Drugs that may impair kidney function	Non-steroidal anti-inflammatory drugs	Selective COX-2 inhibitors have relatively few adverse effects on the kidney	(28, 29)
		No significant difference in the risk of kidney injury between COX-2 inhibitors and non-selective COX inhibitors	(30)
		NSAIDS can cause drug-induced acute interstitial nephritis (AIN)	(31–33)
	ACEI and ARB	Perioperative treatment with ACEI/ARB increases the incidence of postoperative AKI	(34–36)
		Absolute risk of perioperative AKI reduced with ACEI/ARB	(37)
	Antibiotics	Aminoglycosides can cause renal tubular toxicity	(38)
		Vancomycin is most likely to produce nephrotoxicity through increased reactive oxygen species and oxidative stress	(39)
		Fluoroquinolones were graded in order of nephrotoxicity as ciprofloxacin, moxifloxacin and levofloxacin fluoroquinolones can cause AIN	(40, 41)
		High-dose cephalosporin treatment causes proximal tubular necrosis and renal insufficiency in rats	(42)
Intravenous (arterial) injection of contrast media		The prevalence of CI-AKI is 2% in the general population but increases to 20–40% in high-risk patients	(43)
		There was no significant difference in the incidence of AKI between the contrast and control groups	(44)
		Intra-arterial contrast injection is more nephrotoxic than intravenous use	(45)
		No significant difference in AKI incidence with vs. without PCI in STEMI patients	(46)
Special surgical interventions	Heart surgery	Higher incidence of AKI after heart valve surgery with increased subsequent dialysis dependence and in-hospital mortality	(47)
	Liver transplantation	The incidence of perioperative AKI is high, and the occurrence and progression of AKI affect the short-term and long-term survival of the graft	(48)
	Abdominal aortic aneurysm surgery	The operation can increase the risk of perioperative AKI	(49)
		Severity of postoperative AKI after open repair is independently associated with increased in-hospital mortality in patients with postoperative AKI	(50)
	Pulmonary endarterectomy	The incidence of postoperative AKI is higher in patients with chronic thromboembolic pulmonary hypertension	(51)
Anesthesia	Anesthesia method	Intraoperative MAP consistently <60 mmHg for 20 min and <55 mmHg for 10 min increased the incidence of postoperative AKI	(52)
		Reduced risk of renal failure in patients treated with intraspinal anesthesia compared to general anesthesia	(53)
	Narcotic drugs	Sevoflurane anesthesia reduces kidney injury in small volume liver transplant rats	(54)
		Higher incidence of AKI in patients with sevoflurane than in those receiving propofol	(55)
		Propofol preserves the morphological integrity of the kidney and attenuates AKI in mice undergoing cecum ligation and puncture surgery	(56)
Anemia and the effects of blood transfusion	Anemia	Reduced perioperative hemoglobin concentration is strongly associated with the development of postoperative AKI	(57)
	Blood transfusion	Increased risk of perioperative AKI is directly proportional to the number of red blood cell infusions	(58–60)
Malnutrition		Perioperative nutritional status of patients is closely related to the occurrence of AKI	(61–65)
Hyperglycemia		Hyperglycemia is considered one of the independent predictors of increased mortality and worsened prognosis in perioperative patients	(5)

### Drugs That Impair Kidney Function

Many drugs routinely used in the perioperative period may adversely affect renal function, and it is now believed that up to 25% of cases of severe AKI are triggered by nephrotoxic drugs (Table 2). Non-steroidal anti-inflammatory drugs (NSAIDs) can directly reduce renal blood flow while causing tubular obstruction through crystal deposition and can induce direct cytotoxicity and cell-mediated immune damage mechanisms, leading to acute kidney injury (AKI). Risk factors for the nephrotoxicity of NSAIDs have been analyzed, including CKD, old age, cardiomyopathy with impaired cardiac function, diabetes, diuretic and ACEI therapy, vascular disease, hypertension, and fluid deficiency. Selective COX-2 inhibitors have caused relatively few adverse effects on the kidney, including reduced glomerular filtration rate (GFR), elevated serum creatinine (SCr), and hypertension (28, 29). Even this conclusion is disputed, as it has been suggested that there is no significant difference in the risk of kidney injury between selective and non-selective COX-2 inhibitors (30), and there are objective case reports of serious kidney injury due to COX-2 inhibitors (66). Additionally, NSAIDS are currently considered to be the second- or third-leading cause of drug-induced acute interstitial nephritis (AIN), with an incubation period of up to 6–12 months (31, 32), and although the pathological presentation is relatively mild, with few eosinophil infiltrates and little granuloma formation, it is associated with an increased risk of occurrence with advancing age and is usually considered more likely to progress to chronic kidney disease (32, 33). Therefore, the use of NSAIDs, as one of the measures to reduce the pain burden of surgical patients, must be carefully evaluated to balance their benefits and risks. Especially in the surgical population with underlying renal disease, NSAIDs should be avoided and choose alternative analgesics (including opioids)when necessary (Table 3). However, NASIDS may also be considered along with other analgesic therapies after careful assessment of the patient's status (67).

**Table 3:** Perioperative AKI prevention and management.

**Perioperative AKI prevention**		**Perioperative AKI management**	
Inclusion of renal risk confirmation in preoperative assessment	a) Enhance preoperative specialist evaluation and optimize surgical plan	Early diagnosis	Discovery of AKI etiology Use of biomarkers to supplement serum creatinine and urine output for the early identification of AKI in high-risk patients
	b) Incorporate a multidisciplinary approach to the perioperative care for patients at high risk of AKI		
Minimize intraoperative renal toxin exposure	a) Avoid ACEI or ARB drugs in the perioperative period	Discovery of AKI complications	Correction of disorders of acid-base balance, water and electrolyte imbalance, etc.
	b) Use NSAIDS with caution in the perioperative period, avoid in certain special cases, or choose alternative analgesics	Administration of vasopressors	Maintenance of adequate perfusion pressure (mean arterial pressure > 65 mmHg, systolic pressure > 100 mmHg)
	c) Use the lowest volume of contrast agent that achieves the examination while considering first non-ionic isotonic contrast agent or hypotonic contrast agent	Use of other drugs	Dexmedetomidine: currently considered the most promising effect (in order to ensure the safe use of dexmedetomidine, patients must be carefully selected in clinical practice and the appropriate dose must be determined)
	d) The specific benefits of perioperative hydration are controversial, but studies continue to support this prophylactic measure		Furosemide: guidelines recommend only for correction of fluid imbalances and electrolyte abnormalities in patients with AKI Sodium bicarbonate, dopamine, vasodilators, and natriuretic peptides: not recommended by guidelines at this time
	e) The effectiveness of acetylcysteine and pentoxifylline is still controversy		
	f) Statins may help to reduce the incidence of CI-AKI, but their mechanism of action has not been fully determined		
Intraoperative management and hemodynamic optimization	a) The routine use of hydroxyethyl starch in surgery is not currently recommended for patients with AKI or co-operative risk factors	Nutritional support	Patients with AKI at any stage: ensure an energy intake of 20–30 kcal/kg/day CRRT treatment: provide up to 1.7 g/kg/day of amino acids
	b) Balanced salt solution is recommended to maintain adequate renal perfusion		Non-dialysis patients: provide 0.8–1.0 g/kg/day of amino acids
	c) Guaranteed MAP > 60–65 mmHg (>75 mmHg in chronic hypertensive patients)		
Remote ischemic preadaptation	a) Remote ischemic preadaptation reduced the incidence of major adverse renal events in patients undergoing high-risk cardiac surgery	Renal replacement therapy	Correction of internal environmental disturbances and reduction of excessive fluid load
	b) Remote ischemic preadaptation may promote renal recovery in patients with perioperative AKI		
Drug prevention	c) Statins have been shown to reduce the incidence of perioperative AKI		

Current findings on the use of ACEIs and ARBs during the perioperative period to induce or exacerbate AKI are inconsistent, and the prevailing view now is that perioperative treatment with ACEIs/ARBs increases the incidence of postoperative AKI by the readily accepted mechanism of intraoperative hypotension and renal artery vasoconstriction leading to renal hypoperfusion, both clearly suggesting that preoperative discontinuation of ACEIs or ARBs is a reasonable strategy (34–36). However, the results of Shah et al. (37) showed that the use of ACEIs/ARBs reduced the absolute risk of AKI by 0.09% and the absolute risk of all-cause mortality by 0.35%, and this correlation was mainly evident in patients with preoperative CKD. In contrast, a prospective multicenter study of 949 patients undergoing selective non-cardiac surgery for endpoint events showed no association between perioperative ACEI/ARB use and postoperative AKI events, but it is worth noting the apparent selection bias of this study. In addition, the lack of systematic analysis of the complexity of the hemodynamics of the enrolled patients and the exclusion of urine volume as a diagnostic indicator only by a single indicator of blood creatinine level might have interfered with the final results (68). In conclusion, during the perioperative period, especially on the day of surgery, ACEI or ARB drugs should be avoided as much as possible to protect the kidneys while also reducing the risk of severe hypotension during anesthesia. It is also important to note that repeated use of these drugs should be avoided until the patient awakens postoperatively to minimize the risk of hypoperfusion (Table 3).

In a recent study on antibiotic-induced AKI, partial data from the FAERS (FDA Adverse Event Reporting System) were collected, and the odds ratio (OR) of the antibiotic/AKI association was calculated from 2,042,801 reports. The antibiotics ranked as follows by their ORs: colistin aminoglycosides, vancomycin, methotrexate sulfamethoxazole, penicillins, clindamycin, cephalosporins, macrolides, linezolid, carbapenems, metronidazole, tetracyclines, and fluoroquinolones (69). The nephrotoxicity of these antibiotics can be divided into blood concentration-dependent and time-dependent. β-Lactam antibiotics mainly cause time-dependent nephrotoxicity, while neoquinolones and aminoglycosides mainly cause concentration-dependent nephrotoxicity. There are three main mechanisms by which antibiotics cause nephrotoxicity, namely, dose-dependent tubular necrosis, allergic tubulointerstitial nephritis, and tubular crystalline formation. Excessive concentrations of aminoglycosides in renal tubules cause tubular toxicity by accumulating in lysosomes, Golgi apparatus, and endoplasmic reticulum and binding to membrane phospholipids, altering their turnover and metabolism, leading to phospholipidosis (38). The most likely mechanism of vancomycin nephrotoxicity is attributed, at least in part, to increased reactive oxygen species and oxidative stress, and the risk factors are thought to be high drug concentration (>20 mg/L) or high doses (>4 g/h), concomitant use of other nephrotoxic drugs, prolonged drug treatment (≥7 d, and intensive care status (39). Current cases of AIN caused by quinolones are mainly associated with older fluoroquinolones, fluoroquinolones were graded in order of nephrotoxicity as ciprofloxacin, moxifloxacin and levofloxacin, with relative risks of 2.76, 2.09, and 1.69, respectively (40). In 2014, Muriithi et al. (41) reported a series of kidney biopsy case, it was again confirmed that fluoroquinolones can cause AIN, while follow-up studies also found that early steroid treatment or hydration therapy improved recovery of renal function in patients with drug-induced AIN (70, 71). Similarly, Bird et al. (40) found that the relative risk of AKI caused by treatment with fluoroquinolones was 2.18. However, considering the risk of death due to serious infections, the current study findings do not negate the use of fluoroquinolones, and mainly remind the importance of prescribing in clinical work.

There are more reports related to cephalosporins causing AKI in this century, and in a transcriptomic study analysis by Rokushima et al. (42) on the nephrotoxicity of cephalosporin antibiotics, it was observed that high-dose cephalosporin treatment caused proximal tubular necrosis and renal insufficiency in rats. Mac et al. (72) first reported a case of cefepime causing AIN in 2015. Although cefepime is considered a safe antibiotic from a nephrotoxicity point of view and renal-related adverse reactions are rare, clinicians still need to be aware of the nephrotoxicity associated with the use of cefepime, especially in patients on long-term treatment, who should be monitored closely for renal function parameters. However, there is a lack of large epidemiological findings of cephalosporins alone causing AKI as well as specific controlled studies, a lack of intermediate to high-level evidence to support the association between the two, and the only relevant studies are mostly case reports or adverse renal outcomes after combination with other drugs (69). Therefore, it is still one of the most important drugs for the prevention or treatment of infectious diseases in clinical workup, especially in the perioperative period, and it is believed that more research evidence will be available in the future to support or limit it, as well as to provide more guidance in prescribing doses and drug combinations.

### Contrast Injection

Contrast-induced AKI (CI-AKI) is defined as an increase in BUN and sCr or a decrease in eGFR occurring usually 24–72 h after drug administration (73) (Table 2), but its specific definition is not uniform, which hinders cross-study comparisons. It is now believed that the possible mechanisms of CI-AKI include the following: first, renal hemodynamic changes that cause renal medullary hypoxia; second, direct toxic injury effects of contrast agents on renal endothelial cells and proximal tubular cells (74–76); and third, changes in renal microcirculation due to the release of certain neurohumoral mediators or increased blood viscosity (77). In most patients, CI-AKI is likely to be transient and reversible, although some studies have reported its association with increased long-term patient mortality (78–80). The prevalence of CI-AKI is 2% in the general population but increases to 20–40% in high-risk patients, such as those with diabetes, congestive heart failure, or chronic kidney disease and who are elderly (43), but most studies lack a credible control group or strict adjustment for AKI covariates, leading to infirm conclusions. For example, one study concluded that the incidence of CI-AKI significantly increased with decreasing baseline eGFR values, yet there was no significant difference in the incidence of AKI between the contrast and control groups in any eGFR subgroup, even among patients with eGFR <30 ml/min/1.73 m^2^ (44). Similarly, a clinical evaluation by Wilhelm-Leen et al. (81) of 5.9 million enrolled patients showed that the incidence of AKI was even lower in patients receiving contrast than in controls after adjustment for risk factors.

In recent years, along with the increasing number of patients with vascular lesions receiving endovascular treatment, perioperative contrast nephropathy has been gaining attention. The site of contrast injection may have an impact on the incidence of CI-AKI, and some studies have shown a significantly higher incidence of AKI after coronary angiography than intravenous injection (45), the reason for which may be related to its initial concentration in the renal vascular system, as aortography has demonstrated a greater risk of AKI if the contrast is injected directly into the proximal renal artery. However, the conclusion that arterial contrast injection causes AKI is equally questionable, as demonstrated in the Caspi et al. (46) study. Among STEMI patients, there is no significant difference in the incidence of AKI with or without PCI intervention, and independent predictors of AKI in the PCI treatment cohort included age ≥70 years, insulin-treated diabetes, diuretic therapy, eGFR reduction, cardiac pump failure, and reduced left ventricular ejection fraction, independent of contrast dose. Therefore, it must be noted that CI-AKI is still a controversial topic, and no study has been able to explain it thoroughly, which even questions the rationality of CI-AKI, so it is still an exclusionary diagnosis according to the definition of CI-AKI and needs a more rigorous follow-up study that fully controls for other risk factors or confounders.

Nevertheless, currently in clinical workup, great caution is still needed in the perioperative injection of contrast agents into patients assessed preoperatively to be at risk for renal damage. There are several ways to reduce this risk, including the use of the lowest volume of contrast agent that achieves the examination objectives while first considering non-ionic isotonic contrast or hypotonic contrast (Table 3). In addition, although there is controversy about the specific benefits of perioperative hydration, and there are no firm conclusions about the ideal material, dose, and rate of hydration, many studies still support this prophylactic measure, especially in cardiovascular surgery (Table 3). In addition to. The assessment of cardiac function, 0.9% sodium chloride or isotonic sodium bicarbonate solution is given perioperatively to ensure adequate intravascular volume and to minimize the risk and extent of contrast-induced renal injury (82, 83). This same prophylactic strategy is clearly recommended in the European Society of Cardiology (ESC) guidelines on myocardial revascularization, with specific guidelines recommending the administration of intravenous saline 12 h before and 24 h after the administration of contrast media, with intravenous saline at a rate of 1–1.5 ml/kg/h, especially in patients with GFR <40 ml/min/1.73 m^2^ (84). In terms of drug prevention, there is still controversy regarding the effectiveness of acetylcysteine, which can be supported only partially by evidence (85–87). Similarly, statins may help to reduce the incidence of CI-AKI, but their mechanism of action has not been fully understood (88–91). There are also some reports on pentoxifylline, but most of them focus on animal studies (92, 93), in clinical studies, more emphasis has been placed on its combined effect with drugs such as berberine hydrochloride for drug-induced AKI, and for contrast-associated AKI, although it may reduce creatinine elevation in the short term, its preventive effect cannot be fully confirmed based on the available evidence (94, 95).

### Special Surgical Interventions

Surgery itself is a risk factor for AKI, in particular, some specific types of surgery are associated with an increased risk of kidney injury. A retrospective study of patients undergoing heart valve surgery found a 6.1% incidence of postoperative AKI, new dialysis dependence in 3.1% of AKI patients, death during hospitalization in 48.9%, discharge with recovery of renal function in 42.2%, and permanent dialysis dependence in 8.8% (47) (Table 2). The reasons were the use of preoperative contrast agents, decreased perfusion pressure and reduced pulsatile blood flow during bypass surgery, decreased mean arterial pressure during extracorporeal circulation, exposure to extracorporeal circulation triggering a contact systemic inflammatory response, low temperature or mechanical destruction of red blood cells by extracorporeal circulation, and formation of an obstructive tubular pattern by hemoglobin (96, 97). However, there is no consensus on whether extracorporeal circulation necessarily constitutes a cause of the high incidence of AKI in cardiovascular surgery, previous findings suggest that cardiopulmonary bypass with extracorporeal circulation increases the incidence of AKI and other renal diseases (98, 99), the results of the study by Reents et al. (100) found that non-extracorporeal coronary artery bypass grafting was not associated with a reduction in the incidence or severity of AKI.

One study reported that the incidence of perioperative AKI decreased to 11–68% after the introduction of an end-stage liver disease model scoring system as a basis for liver transplantation matching (101). Hepatic ischemia-reperfusion injury may act as the main pathogenesis of AKI after liver transplantation by driving the systemic inflammatory response. In a study by Hilmi et al. (48), analysis of clinical and laboratory data from 424 liver transplant recipients found that at 72 h posttransplantation, AKI occurred in 221 patients (52%) and concluded that female sex, weight ≥100 kg, high Child-Pugh score, and diabetes mellitus were significantly associated with the occurrence of AKI within 72 h. Additionally, the occurrence and progression of AKI within 72 h after transplantation affected the short- and long-term survival of the graft (48) (Table 2).

Abdominal aortic aneurysm surgery is another surgical operation that has been clearly reported to exacerbate the risk of perioperative AKI. In a study conducted in 2015, 149 patients undergoing open repair of abdominal aortic aneurysms were enrolled and analyzed for postoperative data, 18.8% of whom developed AKI, and the next 33 ± 11 months of follow-up investigation revealed that, although no patients required dialysis treatment, the occurrence of AKI was strongly associated with high mortality and high incidence of cardiovascular disease (49) (Table 2). Whereas, the kind of procedure also affects the incidence of postoperative AKI, the severity of AKI after open repair is independently associated with increased in-hospital mortality in patients with postoperative AKI (Table 2), and major risks include aortic clamping, perioperative hypotension, atherosclerotic embolism, and impaired renal blood flow due to exposure to blood products (50).

Zhang et al. (51) published the first report on a complication study of 123 patients diagnosed with chronic thromboembolic pulmonary hypertension (CTEPH) who underwent pulmonary endarterectomy (PEA). Their data suggested that the incidence of postoperative AKI was 45% (Table 2); that preoperative platelet count, hemoglobin concentration, and duration of deep hypothermic circulation stagnation were independent factors associated with AKI; and that renal protection strategies should be prioritized in the perioperative management of such procedures (51). However, some unknown confounding factors (such as perioperative nephrotoxic drug use) were not explicitly excluded in this study, and there are not enough other relevant studies due to the low incidence of CTEPH, resulting in some limitations of the findings.

### Anesthesia

Intraoperative anesthesia can affect renal function in different ways and increase the risk of perioperative AKI, mainly due to the choice of anesthesia method and drug (Table 2). The study by Sun et al. (52) suggested that intraoperative anesthesia-induced hypotension was closely related to the occurrence of postoperative AKI, and intraoperative MAP consistently <60 mmHg for 20 min and <55 mmHg for 10 min increased the incidence of postoperative AKI. The results of a meta-analysis by Rodgers et al. (53) in 2000 showed that compared with general anesthesia, patients treated with intraspinal anesthesia had a reduced risk of renal failure. However, some studies have also indicated a similar incidence of AKI with epidural anesthesia combined with general anesthesia compared to general anesthesia alone (102), so the specific effects of intraspinal anesthesia on renal function still need to be confirmed by more studies.

In animal studies, sevoflurane anesthesia was shown to reduce renal injury in small-volume liver transplant rats by significantly lowering the 24-h sCr after reperfusion and NGAL concentrations after 2 h reperfusion in rats with sevoflurane (54). However, in human patients, a retrospective study suggested that the incidence of AKI was higher in patients receiving sevoflurane than in those receiving propofol, suggesting that sevoflurane anesthesia may be associated with the development of postoperative AKI (55). In contrast, another domestic animal study suggested that propofol improved survival after cecum ligation and puncture surgery (CLP) in mice, preserved the morphological integrity of the kidney in mice undergoing CLP, and lowered the occurrence of AKI (56). There is a lack of human studies on perioperative anesthetics causing AKI, and more high-level evidence is needed to rule out or confirm this view.

### Anemia and blood transfusion

Walsh et al. (57) concluded that decreased perioperative hemoglobin concentration is strongly associated with the development of postoperative AKI (Table 2). Another study reached the same conclusion, as the postoperative decrease in hemoglobin from baseline was positively associated with the decrease in eGFR, and the OR value was proportional to the degree of hemoglobin decrease (103). The causes are as follows: anemia decreases renal oxygen delivery, especially to the renal medulla, where normal oxygen partial pressure is low; during surgery, the kidneys are more prone to underperfusion, and the important antioxidant function of red blood cells is reduced, aggravating intraoperative renal oxidative stress; and anemia increases the chances of transfusion and aggravates AKI due to transfusion (58).

Previous studies concluded that the increased risk of perioperative AKI is directly proportional to the amount of red blood cell transfusion, and this correlation is particularly evident in anemic patients (Table 2). This finding has been widely confirmed in cardiac surgery, where a variety of blood-sparing measures are recommended (58–60). The causes are that red blood cells become less deformable during storage, while ATP and 2–3DPG are depleted and lose their abilities to produce NO, enhance vascular endothelial cell adhesion, release procoagulant phospholipids and accumulate proinflammatory molecules, free iron, and hemoglobin; stored red blood cells may impair tissue oxygen delivery, promote an inflammatory state, exacerbate tissue oxidative stress, and activate leukocyte and coagulation cascade reactions. Possible mechanisms why anemic patients are more susceptible to transfusion-associated AKI include the following: first, anemic patients are usually more anemic during cardiac surgery, resulting in less oxygen delivery and making the kidneys more susceptible to hypoxic injury; second, some anemic patients will have subclinical nephropathy characterized by increased renal tubular oxygen consumption and oxidative stress, making the kidneys susceptible to acute or chronic damage (104); third, anemic patients have abnormal iron metabolism and a weaker ability to cope with an increased iron load due to multiple transfusions, triggering iron-mediated oxidative kidney injury (105). However, transfusions, like anemia, lack the results of large randomized controlled trial or meta-analyses, and the only conclusions available are mostly complication studies of specific types of surgery, which cannot adequately demonstrate their relationship with AkI in non-cardiac surgery. In addition, it is worth mentioning that in a meta-analysis published in recent years, findings suggest that administering EPO before anesthesia is emerging as an important factor for efficacy. Erythropoietin may have a role in preventing cardiac surgery associated acute kidney injury (CSA-AKI), however, additional high-quality prospective studies are warranted, particularly aimed at the timing and size of the dose (106).

### Malnutrition

Malnutrition is also an important risk factor that increases the incidence of perioperative AKI (Table 2). Nutrition is the basis of cellular and organ function, and malnutrition may worsen the severity of disease by significantly changing renal hemodynamics and renal concentrating capacity (107). Malnutrition in children and adults decreases the glomerular filtration rate, and experimental models show that the renin-angiotensin system, renal prostaglandin secretion and overall renal function are altered in malnourished states, although the exact mechanisms remain incompletely understood (108, 109). This malnutrition can be manifested in several ways, including in the concentrations of albumin, vitamins, electrolytes, minerals, and trace elements. Several studies have confirmed the correlation between low or deficient nutrients and the development of AKI in patients with infectious diseases, cancer, surgery and other causes of critical illness, showing that malnutrition is an independent risk factor and suggesting that following AKI guidelines to prevent and treat AKI by providing nutritional support, especially to children and elderly patients (110–114). In addition, similar views have been proposed in several studies on risk factors assessment for surgery-related AKI, as well as studies on the correlation between preoperative prognostic nutritional index and AKI, it was suggested that perioperative nutritional status of patients is closely related to the occurrence of AKI (61–65).

Nutritional support is also a priority in the follow-up treatment of AKI patients (Table 3). According to the KIDGO guidelines for the nutritional support of AKI patients, an energy intake of 20–30 kcal/kg/day, mainly carbohydrates and fats, should be ensured regardless of the stage of the disease. In the case of CRRT, considering the normal protein metabolic rate and filtration losses, a maximum of 1.7 g/kg/day of amino acids needs to be actively provided to compensate for these losses, while in non-dialysis patients, this value must be 0.8–1.0 g/kg/day, and it is recommended to provide nutrition mostly through the enteral route (5).

### Hyperglycemia

Hyperglycemia is considered one of the independent predictors of increased mortality and worsened prognosis in perioperative patients and should be optimized in perioperative patients. The KDIGO criteria recommend maintaining blood glucose concentrations between 110 and 149 mg/dl in critically ill patients to minimize all-cause mortality, surgical complications and increased risk of AKI due to hyperglycemia in the perioperative period (5) (Table 2).

## Prevention of Perioperative AKI

### Preoperative Assessment of Renal Risk

As part of this process, it is recommended to enrich the details of the assessment, include strictly kidney-related risk factors, integrate and stratify them scientifically, classify the kidney function in a timely manner, identify possible underlying kidney diseases, strengthen contact with anesthesia and kidney specialist physicians, receive professional advice, and provide clinicians with more scientific reference for preoperative guidance, while communicating to the patient team the likelihood and degree of risk of perioperative AKI and optimizing the surgical plan. In addition, it is also possible to incorporate a multidisciplinary approach to the perioperative care for patients at high risk of AKI. However, there are few reports that have taken such an approach, and no specific benefits can be determined (Table 3).

### Intraoperative Management and Hemodynamic Optimization

Perioperative renal protection focuses on maintaining adequate renal perfusion, which is largely dependent on adequate intravascular volume and mean arterial pressure, but fluid supplementation alone may not overcome the effects of hypotension during anesthesia in some patients and may lead to postoperative complications. According to the conclusions of a recent meta-analysis, optimization of intravascular volume and cardiac output may have a positive impact on perioperative renal function in high-risk patients (definition of high risk was based on need of emergent surgery, and/or elective major surgery in patients with risk criteria defined by perioperative scoring system, ASA physical status classification, age>60 years, and preoperative morbidity), at three preoperative, intraoperative or postoperative time points with fluid combination of supplementation and cardiac agents reduces the incidence of renal insufficiency and significantly reduces mortality (115), but this procedure requires careful monitoring of volume status to avoid volume overload, and in terms of specific fluid selection, balanced salt solutions are more recommended to avoid the risk that AKI that is exacerbated by perioperative saline overinfusion will lead to hyperchloremia (116). While the use of colloidal solutions is controversial, and the routine use of hydroxyethyl starch in surgery is not currently recommended for patients with AKI or co-operative risk factors (117–119). As for the threshold of mean arterial pressure, several studies have confirmed that even short-term hypotensive states can damage the kidneys, so ensuring MAP >60–65 mmHg (>75 mmHg in patients with chronic hypertension) is recommended to prevent AKI (52, 120) (Table 3), and a more individualized approach to intraoperative arterial pressure management should also be taken in this process.

### Remote Ischemic Preadaptation

Remote ischemic preadaptation is an experimental approach to provide organ protection with short cycles of harmless ischemia and reperfusion applied to the arm or leg, a pathway thought to drive the stabilization of transcription factors such as hypoxia-inducible factor (HIF1a or HIF2a), a transcriptional program that mediates the release of soluble mediators (IL-10, adenosine, circulating nucleotidases) from ischemic muscle into the body circulation, thereby providing protection to remote organs (e.g., the heart or kidney) (121) (Table 3). Zarbock et al. (122) conducted a large, multicenter, randomized, double-blind clinical trial in 2015, which suggested that remote ischemic preadaptation significantly reduced the incidence of major adverse renal events at 90 days in patients undergoing high-risk cardiac surgery compared to controls, while suggesting that remote ischemic preadaptation may promote renal recovery in patients with perioperative AKI. Similarly, the results of two recent meta-analyses confirm this view (123, 124). However, like others, this conclusion still faces contrary opinions. Hausenloy et al. (125) included a controlled study of a total of 1,612 patients undergoing cardiac surgery in 30 centers with an observation period of 12 months and showed that the difference in the incidence of major endpoint events between patients in the remote ischemic preadaptation and control groups was not statistically significant, including the incidence of AKI, duration of ICU care, and length of hospital stay. More recently, data from a meta-analysis on 3,660 patients in 43 RCTs show a similar conclusion that remote ischemic preadaptation reduced cardiovascular events after non-cardiac surgery, but had no significant advantage for the incidence of AKI and all-cause mortality (126). Therefore, further studies are still needed to collect relevant evidence and validate the specific effect of remote ischemic preadaptation in perioperative AKI prevention.

### Drug Prevention

Statins have potential anti-inflammatory and antioxidant effects and have been shown to reduce the incidence of perioperative AKI, the risk of RRT, and 14-day mortality in a clinical study enrolling 200,000 patients (127) (Table 3), but there are no consistent findings in several studies related to cardiac surgery (128, 129), and in recent years the preventive effects of statins on contrast nephropathy (CIN-AKI) have been supported by the results of several studies (91), so the general recommendation on statins for AKI is currently uncertain because of the lack of evidence from prospective trials.

## Perioperative AKI Management

### Early Diagnosis

A detailed bedside examination is performed to look for all possible etiologies that may precipitate perioperative AKI (Table 3), mainly from the prerenal, renal and postrenal perspectives and to rule out causes such as urinary tract obstruction as quickly as possible with the help of imaging. Blood creatinine values and urine volume are also closely monitored to keep track of changes dynamically, to quickly assess the patient's volume status, and to check whether the patient has sufficient vascular volume reserve or possible overload. In addition, we suggest the use of biomarkers to supplement serum creatinine and urine output for the early identification of AKI in high-risk patients (Table 3), especially target molecules that have been approved for use in some jurisdictions, such as NGAL, L-FABP, TIMP-2, and IGFBP7. However, this is not easy. On the one hand, the source of some biomarkers is not clear, and it is also difficult to determine the cut-off threshold of each marker for different races. On the other hand, the complexity of perioperative patients also interferes with the diagnostic performance of markers.

### Discovery of AKI Complications

Timely detection and management of various complications of perioperative AKI (Table 3), including disorders of acid-base balance, water and electrolyte imbalance (hyperkalemia, volume overload, acidosis, etc.), and consideration of reduced fluid infusion in some patients are needed to maintain in/out balance, but special attention needs to be paid to the estimation and calculation of hidden fluid losses during this period.

### Administration of Vasopressors

The protection of renal function depends to a large extent on the maintenance of renal perfusion, and the maximum benefit of treatment after renal damage in high-risk surgical patients can be achieved by maintaining hemodynamic stability. In cases where fluid resuscitation is ineffective or can only be maintained briefly, vasopressors can be administered to maintain adequate perfusion pressure (mean arterial pressure > 65 mmHg and systolic pressure > 100 mmHg (130, 131) (Table 3). In addition, in patients with moderate to severe ventricular insufficiency, concurrent administration of positive inotropes, and fluid therapy may be attempted. However, in patients with complex conditions, the best approach is to provide a highly dependent environment that allows for optimal monitoring.

### Use of Other Drugs

Due to the heterogeneity of AKI, identifying a single therapy that will benefit all patients is challenging, and numerous drugs are being studied in clinical trials, but the overall results remain less than promising (Table 3). Dexmedetomidine, a highly selective α-blocker, has been shown to have renoprotective effects in several cardiac surgery studies and is thought to act mainly by reducing norepinephrine release, improving hemodynamic stability, and maintaining myocardial oxygen supply balance, thereby significantly reducing the incidence of AKI, especially in patients with normal or mildly impaired preoperative renal function (132, 133). The most recent meta-analysis in 2021 confirmed that dexmedetomidine had a renoprotective effects after surgery, with NGAL levels reduced and creatinine clearance significantly increased in patients treated with it (134). Unfortunately, there are no clear recommendations on treatment strategies, including in the KIDGO guidelines. Therefore, we suggest that in order to ensure the safe use of dexmedetomidine, patients must be carefully selected in clinical practice and the appropriate dose must be determined.

Furosemide is a widely used diuretic in clinical practice, and data from some studies suggest that it may improve the balance of oxygen supply and demand in the renal medulla by inhibiting _Na−K−Cl2_ cotransporter activity, increasing prostaglandin production and blood flow, and preventing tubular obstruction due to endothelial cell shedding. However, clinical studies based on this hypothesis have not yielded positive results and have concluded that it should not be used for the routine treatment of AKI (135, 136). Even so, the KIDGO guidelines, mainly the Japanese AKI guidelines and the NICE (National Institute for Health and Clinical Excellence) guidelines, still recommend furosemide for the correction of fluid imbalances and electrolyte abnormalities in patients with AKI (Table 3).

Other drugs, including sodium bicarbonate, dopamine, vasodilators, and natriuretic peptides, have been clearly demonstrated in multiple studies to have no AKI prevention or treatment benefit and are not included in guideline recommendations. Later studies may provide evidence to the contrary, but at least at this stage, dexmedetomidine has the most promising effect (Table 3).

### Renal Replacement Therapy

There is no positive evidence that renal replacement therapy has a positive effect on the development of perioperative AKI, but replacement therapy itself can correct internal environmental disturbances and reduce excessive fluid load and can be used in a timely manner at the onset of AKI to reduce symptoms while buying valuable time for comprehensive treatment in other areas (Table 3). In particular, continuous renal replacement therapy is recommended for patients with complex conditions and hemodynamic instability. In addition, to decide on the use of anticoagulants, detailed information about the patient's bleeding history and major surgery history must be obtained to fully determine the patient's coagulation function, and heparin can be avoided or replaced with topical citrate or argatroban anticoagulation to avoid the risk of severe bleeding when conditions permit (137). The ideal mode of renal replacement therapy, its timing and the duration of initiation of therapy are still under debate. A randomized controlled study of 620 patients conducted by Gaudry et al. (138) in 2016 suggested no significant difference in mortality between patients in the early and delayed strategy groups, with patients in the early strategy group mostly starting therapy within 6 h. In contrast, other clinical studies have drawn different conclusions, the representative report being a randomized controlled study of 231 patients conducted by Zarbock et al. (139) in 2019, which found that the early strategy significantly reduced mortality at 90 d compared to the delayed strategy.

## Concluding Remarks

The pathophysiologic mechanisms by which perioperative AKI occurs are complex and varied, but the outcomes all increase the risk of patient death, and the effects of even mild AKI are severe, being correlated with a negative prognosis and ongoing increased mortality. Most cases of AKI occurring in the perioperative period are associated with relative renal hypoperfusion and/or renal damage by nephrotoxins and are less associated with primary renal disease. The likelihood of serious, long-term, progressive consequences can be reduced and the perioperative prognosis improved by following reasonable strategies for early avoidance of perioperative AKI risk factors and enhanced perioperative patient management, as well as a rapid response to and early management of AKI when it occurs.

## Author Contributions

All authors listed have made a substantial, direct, and intellectual contribution to the work and approved it for publication.

## Funding

The work was supported by the Science and Technology Project of Beijing, China (Z191100006619001), and the Chinese National Natural Science Foundation (No. 81670694).

## Conflict of Interest

The authors declare that the research was conducted in the absence of any commercial or financial relationships that could be construed as a potential conflict of interest.

## Publisher's Note

All claims expressed in this article are solely those of the authors and do not necessarily represent those of their affiliated organizations, or those of the publisher, the editors and the reviewers. Any product that may be evaluated in this article, or claim that may be made by its manufacturer, is not guaranteed or endorsed by the publisher.
